# Antennal lobe representations are optimized when olfactory stimuli are periodically structured to simulate natural wing beat effects

**DOI:** 10.3389/fncel.2014.00159

**Published:** 2014-06-12

**Authors:** Benjamin Houot, Rex Burkland, Shreejoy Tripathy, Kevin C. Daly

**Affiliations:** ^1^Department of Biology, West Virginia UniversityMorgantown, WV, USA; ^2^Centre des Sciences du Goût et de l’Alimentation, Université de BourgogneDijon, France; ^3^Center for the Neural Basis of Cognition, Carnegie Mellon UniversityPittsburgh, PA, USA

**Keywords:** olfaction, temporal coding, oscillations, synchrony, active sensing, sniffing, odor representation

## Abstract

Animals use behaviors to actively sample the environment across a broad spectrum of sensory domains. These behaviors discretize the sensory experience into unique spatiotemporal moments, minimize sensory adaptation, and enhance perception. In olfaction, behaviors such as sniffing, antennal flicking, and wing beating all act to periodically expose olfactory epithelium. In mammals, it is thought that sniffing enhances neural representations; however, the effects of insect wing beating on representations remain unknown. To determine how well the antennal lobe (AL) produces odor dependent representations when wing beating effects are simulated, we used extracellular methods to record neural units and local field potentials (LFPs) from moth AL. We recorded responses to odors presented as prolonged continuous stimuli or periodically as 20 and 25 Hz pulse trains designed to simulate the oscillating effects of wing beating around the antennae during odor guided flight. Using spectral analyses, we show that ~25% of all recorded units were able to entrain to “pulsed stimuli”; this includes pulsed blanks, which elicited the strongest overall entrainment. The strength of entrainment to pulse train stimuli was dependent on molecular features of the odorants, odor concentration, and pulse train duration. Moreover, units showing pulse tracking responses were highly phase locked to LFPs during odor stimulation, indicating that unit-LFP phase relationships are stimulus-driven. Finally, a Euclidean distance-based population vector analysis established that AL odor representations are more robust, peak more quickly, and do not show adaptation when odors were presented at the natural wing beat frequency as opposed to prolonged continuous stimulation. These results suggest a general strategy for optimizing olfactory representations, which exploits the natural rhythmicity of wing beating by integrating mechanosensory and olfactory cues at the level of the AL.

## INTRODUCTION

Sensory systems, regardless of modality, must rapidly resolve an ever-changing sensory field. To facilitate perception, active sampling behaviors periodically structure sensory input as well as subsequent physiological responses. The importance of periodic sensory input produced by active sampling behaviors has been observed across sensory modalities, including for example, vibrissal whisking in the somatosensory system (Carvell and Simons, 1990) and micro saccades in the visual system (Ditchburn and Ginsborg, 1952; Martinez-Conde et al., 2006). These studies suggest that perception is therefore impacted by the active behavioral control of the interaction between the sensory structure and the environment it senses (Martinez-Conde et al., 2004; Moore, 2004).

As with somatosensory and visual systems, primary olfactory networks are responsive to the temporal structure of sensory input. Temporal structure arises from the stochastic nature of odor plumes and trails. Detailed characterization of odor plumes indicate that they are discontinuous with a temporally complex filamentous structure that results in exposures that range from <100 ms to more than a second; however, this is dependent on environmental features (Mafraneto and Carde, 1994; Murlis et al., 2000). Nevertheless, as with other sensory modalities, animals often impose temporal structure onto the olfactory sensory array using active sampling behaviors (Halpern, 1983). These behaviors include antennal flicking in crustaceans (Schmitt and Ache, 1979; Daniel and Derby, 1991; Leonard et al., 1994; Goldman and Patek, 2002; Mead et al., 2003) and in some insects (Hillier and Vickers, 2004), sniffing in mammals (e.g., Teghtsoonian and Teghtsoonian, 1982; Laing, 1983; Youngentob et al., 1986, 1987), tongue flicking in snakes (Halpern and Kubie, 1980), and zigzagging flight in a wide variety of insects (Kennedy and Marsh, 1974; Willemse and Takken, 1994; Carde, 1996; Fadamiro et al., 1998). Key to these behaviors is the periodic or rhythmic nature of sensory sampling that they impose, but we note that at least some of these behaviors can also be influenced by the details of the plume structure (Willis and Baker, 1984; Murlis et al., 2000). This sampling is accomplished by moving the sensory epithelium through the olfactory environment (as in flicking) or moving the olfactory environment over the sensory epithelium (as in sniffing). The intermittency of exposure is thought to result in more distinctive neural representations and reduce sensory adaptation, thereby enhancing overall odor perception (Mainland and Sobel, 2006; Schoenfeld and Cleland, 2006).

Plume tracking insects, such as moths, require intermittent, as opposed to continuous, odor exposure in order to maintain their upwind flight toward the source (Kennedy et al., 1981; Willis and Baker, 1984). It has long been argued that wing beating can enhance plume tracking performance (Schneider, 1964). For example, in the flightless silkworm moth *Bombyx mori*, males still vigorously beat their wings as they track pheromones to their sources; removal of the wings results in a loss of ability to find an odor source (Obara, 1979). More recently, modeling and anemometric studies have shown that the wing beat imposes a dynamic and periodic structure to the airflow around the antennae in *Manduca sexta* (Sane, 2006; Sane and Jacobson, 2006), which tracks plumes on the wing. In the flightless silkworm moth *B. mori*, the effect of wing fanning is a predicted 560x increase in air velocity through gaps between antennal olfactory sensilla (Loudon and Koehl, 2000). Furthermore studies of wing kinematics indicate that odor guided and hovering flight brings the wings closer to the antennae (Willmott and Ellington, 1997a, b), thereby increasing the wings effect on airflow around the antennae. Thus wing beating, like sniffing or antennal flicking, directly influences the interaction of antennal receptors with the olfactory environment by driving a rapid and oscillating airflow over the olfactory epithelium, thereby imposing a periodic temporal structure upon sensory input and processing.

Spike patterning of the principle output neurons (projection neurons or PNs) of the antennal lobe (AL) have long been known to vary predictably with the temporal features of the stimulus on a reasonably rapid timescale on the order of 50 ms (Christensen et al., 1998; Heinbockel et al., 1999; Vickers et al., 2001). Consistent with active sampling, more recent results demonstrate that both peripheral (Vickers et al., 2001; Bau et al., 2002; Vickers, 2006; Tripathy et al., 2010) and central nervous system structures (Tripathy et al., 2010) are able to encode odor stimuli presented with flow dynamics of the natural wing beat frequency. During tethered flight, electroantennogram amplitudes increase by 2–3 times relative to when the wings are at rest, suggesting that the flow dynamics established by wing beating strongly influences olfactory input (Bau et al., 2005). Furthermore, the moth olfactory system both tracks (Tripathy et al., 2010) and integrates (Tabuchi et al., 2013) olfactory information across very brief temporal windows, such as those produced by each wing beat. These more recent findings indicate that the olfactory system of the moth can resolve temporal features more rapidly than previously thought and are consistent with the hypothesis that olfactory systems are tuned to sample inputs within a timeframe defined in part by the innate behaviors that influence sensory exposure.

Our previous studies establish the ability of the olfactory system of *Manduca* to track the temporal dynamics of periodic stimuli up to and beyond the maximum wing beat frequency (~28 Hz) and that is associated with an increase in behavioral sensitivity when odors are presented as pulse trains (Tripathy et al., 2010; Daly et al., 2013). However, these studies only quantified the frequency response of individual units and subthreshold local field potential (LFP) oscillations to single odors. Thus, our previous work could not determine whether pulse tracking was odor dependent or whether these temporally structured stimuli impacted AL population level representations of odors as measured using population analytic techniques. Are neural representations for odor, the spatiotemporal patterns of neural activation/inactivation, more distinct when odor is presented in a manner that simulates the effect of wing beating? This is an important question as many olfactory processing studies employ relatively prolonged continuous stimuli and these stimuli underlie prolonged temporal responses within the AL.

Therefore, the aim of the current study was to understand how periodic olfactory stimulation, on the rapid timescale induced by wing beating, shapes the spatiotemporal structure of odor-driven AL representations in the moth, *M. sexta*. Specifically, we sought to test the hypothesis that odor stimulation at the natural sampling frequencies improves odor representations. We first determined the impact of additional stimulus features on pulse tracking, including odorant molecular features, odor concentration and stimulus duration. We then assessed whether periodic stimulation affects the phase relationships of neural unit spiking with LFPs, which have previously been thought to require prolonged continuous stimulation to emerge and is thought to be a spike time synchronizing mechanism necessary for fine odor discrimination (Laurent, 2002). Finally, we asked whether representations for different odors are more statistically separated or distinctive, relative to prolonged continuous stimuli, when odor is presented as brief and rapid pulse trains. We predicted that these brief and rapid pulse trains of odor, which arise in nature as a consequence of both the intermittent nature of odor plumes and the superimposed periodic effects of the wing beat, produce more distinctive odor representations relative to continuous odor delivery.

## MATERIALS AND METHODS

### SUBJECTS

*Manduca sexta* moths were raised in the laboratory using standard techniques (Bell and Joachim, 1976). At pupal stage 17, moths were individually placed into paper bags and kept in an incubator (Percival Scientific; I66VLC8) with a 16:8 light/dark photoperiod and constant temperature (25°C) and relative humidity (75%). All experiments were performed on adult male moths that were between 5 to 7 days post-eclosion.

### PROCEDURES

#### Surgical preparation

The surgical methods used have been previously described in detail (Daly et al., 2004a, b). To secure the head for dissection and recording, moths were placed into a 6 cm long by 12 mm inner diameter copper tube. The head was held in place with soft dental wax. To expose the brain, a ~2 mm^2^ section of the center of head capsule was first excised. Next, a second portion of the head capsule, directly caudal to the first, was sectioned with cibarial pump muscles attached and intact; this portion of head capsule was moved forward into the previously opened notch then glued in place with superglue. This approach provides access to the brain and specifically the AL while keeping the moth fully intact and functional. The brain was then superfused with physiological saline (Heinbockel et al., 1998). The trachea delineating the boundary of the AL from the rest of the deutocerebrum was gently removed to facilitate electrode positioning and penetration. The intrinsic muscles of the left antenna were cut to enhance stability. Two minuten pins were imbedded in the wax near the eyes to restrain the left antenna.

#### Olfactory stimulation

**FIGURE 1:**
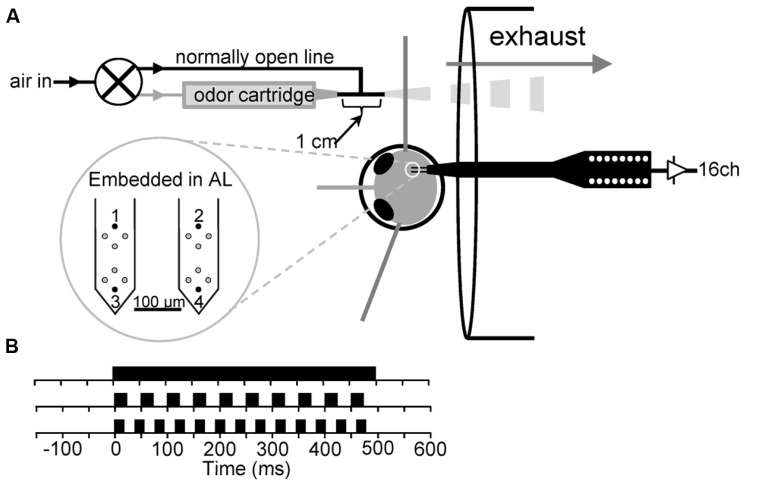
**(A)** Schematic of experimental preparation. **(B)** Schematic of the 500 ms continuous stimulus (top) and the 500 ms, 20 Hz (middle) and 500 ms, 25 Hz (bottom) pulse trains. Note that a 50% on/off duty cycle was used for both pulse trains.

A schematic of the olfactometer with respect to the experimental preparation is shown in **Figure 1**. Olfactory stimuli were delivered as described previously (Tripathy et al., 2010). Prepared moths were placed 2 cm in front of a 6 cm diameter exhaust vent to remove spent odor from around the antenna. Exhaust flow was measured at ~0.7 m/s at the position of the antenna using a Fisher brand hotwire anemometer. Stimulus delivery was controlled by a three-way odor control valve (Lee Co.: LFAA1200118H). Air for the olfactometer was supplied via a central airline, passed through a 500-cc Drierite cartridge to extract moisture (Indicating Drierite; Drierite: 23025) and then through 500-cc of active charcoal (Sigma-Aldrich: C3014) in a modified Drierite cartridge. Cleaned air was then passed through an adjustable glass ball flow meter (Cole-Parmer: 1-010293). From the flow meter, the air was rehumidified by passing it through an aquarium aerator stone in deionized water. The rehumidified air was then fed into the three-way valve, over the antennae and into the exhaust vent. The nozzle of the olfactometer was a nylon barbed T-fitting with a 1.6 mm ID. One of the three ends of the T served as the odor delivery nozzle and was placed ~2–3 mm from the antenna to deliver the odor. The other two openings of the T-fitting received either clean air from the normally open line of the odor control valve, or air from the normally closed line, which passed through an odor cartridge. Thus, air was constantly flowing through the system, over the antenna and into the exhaust. The airflow velocity from the olfactometer at the antenna was set to 0.3 m/s, which is within the range of odor guided flight (Willis et al., 2013).

The odor cartridges were made from borosilicate glass tubing with air tight nylon fittings on each end that connected to the normally closed line on one end and the T-fitting on the other. The completed cartridge had an inner volume of 1.7 ml. An aliquot of 2 μl of odor was deposited on a strip of #1 Whatman filter paper and inserted into the cartridge. Depending on the experimental details, odorant may have been undiluted (i.e., neat) or diluted in mineral oil. A glass cartridge containing only a strip of filter paper was used as a blank control.

Custom scripts run within the Neuralynx data acquisition environment controlled the three-way valve. Timestamps for valve actuation were recorded as well as the odorant used. Odorants were delivered in a random sequence. Odor was delivered to the antennae either as pulsed stimuli, or as a continuous stimulus; the duration of stimuli were experiment specific, but we always maintained 10 s inter-trial intervals. Finally we always used a fixed 50% (on:off) duty cycle, thus the individual odor pulses of a pulse train were 25 or 20 ms for the 20 and 25 Hz pulse trains, respectively, and each odor pulse was followed by an inter-pulse interval of the same respective duration.

#### Electrophysiology

16-channel silicon microprobes (2 × 2 TET; NeuroNexus Technologies, Ann Arbor, MI, USA) were used for all multiunit and local field recordings (**Figure 1**). Electrodes on each shank are arrayed in and used as tetrodes with an interelectrode distance of 25 μm. Probe insertion into the AL was performed via an HS6-3 motorized micromanipulator controlled by a MCL-3 electronic controller (World Precision Instruments, Sarasota, FL, USA). Probe placement was performed using a dissecting microscope. Both shanks were inserted along the caudal boundary of the AL and the animal was oriented such that the probes impaled along this boundary. The mean depth of probe tips was 545 μm. Probe signals were buffered by a 27 channel head stage preamplifier (HS 27, Neuralynx, Bozeman, MT, USA), which was connected to a 32-channel amplifier array of the Cheetah data acquisition system (Neuralynx, Bozeman, MT, USA). Unitary event data from the 4 tetrode sites were band pass filtered at 600–6000 Hz and sampled at 33.3 kHz per channel (12 bit). Additionally, the signals of the upper- and lowermost electrodes on each of the two shanks (**Figure 1** numbered electrodes) were continuously sampled at 11.1 kHz to provide LFP recordings. These signals were hardware bandpass filtered from 1 to 125 Hz online. In some cases we later bandpass filtered LFPs from 5–80 Hz using a custom windowed sync filter combined with a 60 ± 2 Hz notch filter to remove line noise.

#### Spike sorting

Spikes were sorted offline using the KustaKwik toolbox for Matlab (Version 3.5.A.23; AD Redish). This is a semi-automated spike-separation algorithm that identifies clusters of spikes in a 12-dimensional waveform feature space. In this case spike energy and the first two principal components of the waveforms were used. KustaKwik processes data through an expectation–maximization fit of *n* Gaussians to the data to determine a possible set of putative clusters. Final cluster selection was manually performed and based on different criteria: their waveform profiles across the four recording sites must be specific and stable across the entire experiment for all recording sites and contain ≤5% of spikes with inter-spike interval (ISI) histograms of 2 ms or shorter (i.e., contamination). At this point, we defined each cluster of spikes as one neural “unit.”

#### Experimental protocols

The primary goal of this study was to characterize the ability of the AL to produce neural representations for odor that was either pulsed to replicate wing beating or as continuous stimuli. We furthermore characterized responses as a function of differences in the molecular features of odors, their concentration and stimulus duration. To achieve these goals we tested moths in three groups.

In the first group of moths, we presented odors as: (1) continuous stimuli; (2) pulsed at 20 Hz; and (3) at 25 Hz. All stimuli were 500 ms in duration and repeated 10 times. This stimulus duration was based upon the previously characterized “burst length” duration of odor plumes (Murlis et al., 2000) and the previously characterized mean inter-turn duration during upwind flight of *M. sexta* (Willis and Arbas, 1991), thus 500 ms approximates the equivalent of a single pass through a natural plume and hence the longest typical exposure during plume tracking flight. We used two homologous series of ketones and alcohols (see **Table 1** for a complete list including source, purity, density, and name abbreviations) based on their extensive prior psychophysical and physiological characterization (Daly et al., 2001a, b, 2004b, 2013; Mwilaria et al., 2008; Staudacher et al., 2009). For this experiment, undiluted (neat) odorant was used. A total of 15 recordings (*N* = 150 neural sorted units) were made of sufficient quality across the experimental recording period to be analyzed further.

**Table 1:** Panel of odorants used in experiments.

Odorant	Abbreviation	Source	Purity (%)	Density	Molecular mass
***Alcohols***					
1-Hexanol	A6	Sigma	97	0.861	102.17
1-Heptanol	A7	Sigma	99	0.822	116.20
1-Octanol	A8	Sigma	99	0.820	130.23
1-Nonanol	A9	Fluka	98	0.880	144.25
1-Decanol	A10	Sigma	97	0.890	158.28
***Ketones***					
2-Hexanone	K6	Sigma	98	0.810	100.16
2-Heptanone	K7	Sigma	99	0.815	114.18
2-Octanone	K8	Sigma	98	0.820	128.21
2-Nonanone	K9	Sigma	99	0.821	142.24
2-Decanone	K10	Sigma	98	0.824	156.26

*Listing of odorant, text abbreviation, source, purity and density and molecular mass.
*

Some of the more volatile odors in our odor panel are likely to be more concentrated when presented in an undiluted format and therefore elicit stronger responses, which may be more difficult for the moths olfactory system to track, because for example high concentration stimuli may require more time to clear from the system (i.e., a longer decay time constant). Therefore, a second group of 5 moths (*N* = 78 units) were stimulated with three ketones, K6, K7, and K8. In this case, each odorant presented across six concentrations from 0.01 to 100 μg/2 μl in log step dilutions in mineral oil (Fisher Scientific; O121-1). The initial dilutions were calculated based on correcting for the different molecular densities of each odorant in order to produce initial dilutions of 100 μg/2 μl for all odors, which was then further diluted in log steps. This dilution range was selected because behavioral studies indicate that moths can detect these particular odors across this range of concentrations (Daly et al., 2007). Stimuli were presented as both continuous stimuli and as 20 Hz pulsed stimuli as described above. In this group however, stimuli were presented for 2 s in order to characterize how coherently (or phase locked) the AL acted as a network to pulsed stimuli within the context of relatively prolonged stimuli required for temporal coding mechanisms (Laurent, 2002).

Finally, prolonged exposure to odor typically results in attenuation of antennal responses to odor (Daly et al., 2007) and loss of behavioral responses in wind tunnel assays (Baker et al., 1985). These effects are due in part to sensory adaptation as prolonged continuous stimulation and furthermore do not represent typical exposure in the natural environment (Murlis and Jones, 1981). Thus we sought to characterize the ability of the AL to produce persistent responses to pulse trains as a function of increasing stimulus duration where brief interstitials of clean air between each odor pulse allows the olfactory system time to recover. Thus in this final group of 16 moths (*N* = 168 sorted units), we varied the stimulus duration from 0.5 to 3 s in 0.5 s steps (six steps total). Stimuli were presented as both continuous and 20 Hz pulsed stimuli. We used only two odors (K6 and K10) in order to present odor across a relatively broad range of durations while minimizing adaptation across stimuli. Durations were sequentially presented in both decreasing and increasing fashion. Each stimulus/duration was repeated 5 times and undiluted odors were used. Finally, in all groups of moths we presented stimulus matched blank odor cartridges.

Our final goal was to determine if odors presented in this manner produce more distinctive neural representations. We specifically sought to determine if: (1) pulsed and continuous odor representations for the same odor differed; and (2) if pulsed stimuli produced more distinctive representations than continuous stimuli. In order to characterize the degree to which the neural ensemble response changed as a function of stimulation protocol (i.e., a within odor comparison of pulsed versus continuous) or the molecular features of the odors (i.e., a between odor comparison within each stimulation protocol), we calculated the Euclidean distance (ED) between population responses (Chapin and Nicolelis, 1999; Stopfer et al., 2003; Daly et al., 2004b; Hallem and Carlson, 2006; Staudacher et al., 2009). Using pooled data from the first group of moths (*N* = 150 units), where the entire panel of odors was presented, we calculated an ED by time.

### ANALYSIS

#### Classification of unitary responses to pulsed odor

The characterization of pulse tracking responses (i.e., AL responses that are temporally structured in a manner that reflects entrainment to the temporal structure of the odor pulse train) from unitary spiking was based on the power spectral density (PSD) analysis implemented using custom scripts written in MATLAB. This analysis was performed on all datasets. Spike time data from individual neural units were binary interpolated to a constant sampling rate of 33.3 kHz. Welch’s method was then applied by first segmenting the filtered signal into eight blocks of equal length (where the overlap between each block generated was 50%), then averaging their Fourier transforms (Tripathy et al., 2010; Daly et al., 2011). This analysis provides a frequency resolution of 0.16 Hz. Pulse tracking responses were identified based on measures of power integrated around a frequency window of 4 Hz centered on the pulse train frequency (i.e., 20 or 25 Hz). The identification of pulse tracking responses was determined by statistically comparing the mean integrated power at the pulsing frequency from the continuous stimuli (which represents intrinsically produced oscillatory power) and pulsed stimuli. Wilcoxon rank sum test (*p* < 0.05) was used for both an omnibus test for significant integrated power for each unit across odors as well as within individual odors. Units with a significant increase in integrated power at the pulsing frequency were identified as pulse tracking. The results of this preliminary statistical analysis could then be further analyzed parametrically to determine which parameters of the stimulus affected the amount of pulse tracking power elicited from individual units (see below).

#### Parametric analysis of integrated power

In order to determine which factors of the stimulus affect measures of pulse tracking power. General linear modeling (GLM) was performed on the calculated integrated power because this method handles both categorical and continuous variables and their interactions and hierarchically partitions variance components allowing for more stringent tests of significance for key variables. These statistical analyses were implemented in SAS using a significance level of *p* < 0.05. In cases such as carbon chain length where a linear or curvilinear function could be used to describe the variable as a continuous function, linear and polynomial regression functions were calculated for mean values and inset in the Figures below. In all cases we present the most parsimonious function that best fits the data based on comparison of *R*^2^ values. Note that although blank responses are displayed in series, they were not used in the calculation of any inset regression function.

#### Cross correlations and cross spectral density (CSD) analyses

In order to quantify LFP-unit relationships, cross correlations between LFP oscillations and unit spiking were calculated for each unit and individually in response to each stimulus using one of the LFP recordings to provide an indication of phase locking (Laurent and Davidowitz, 1994; Laurent and Naraghi, 1994; Laurent et al., 1996; Stopfer et al., 1997; Daly et al., 2011). The LFPs from electrode site 1 (**Figure 1**, top left electrode) was used as the reference for all cross correlations and a 1 ms binning window was used on a 4 s sampling window. Data were then sub sampled from two non-overlapping peri-stimulus epochs; a spontaneous epoch (-2.0 to 0.0 s) and an odor-driven epoch (0.0–2.0 s), where 0.0 s represents stimulus onset. We performed this analysis on the group receiving odor at different concentrations. Here we selected for analysis responses to the blanks and two of the odor dilutions (0.1 and 100 μg/2 μl).

Next, to quantify the periodic temporal structure in the cross correlation, we calculated the CSD for each cross correlation (Goldberg and Brown, 1969; Galán et al., 2006; Daly et al., 2011). The CSD analysis quantifies the frequency content of any periodic temporal structuring of the cross correlation between LFP and unit. Thus the CSD establishes the frequency and magnitude of any periodic phase relationship. The CSD was a calculated individually for each cross correlation using the same methods as described above for the PSDs. CSD power was parametrically compared (again using GLM) to determine the relative contribution of temporal structuring driven by the temporal dynamics of the pulse trains as opposed to the intrinsic properties of the network. Statistical consistencies in the CSD measures between the pulsed and continuous stimuli as well as spontaneous activity inform us about the intrinsic properties of circuit dynamics. Significant differences in the CSD measures between pulsed stimuli and the continuous stimuli, on the other hand, establish whether the temporal correlations (i.e., phase locking) are largely dependent on the stimulus parameters.

#### Euclidean distance analysis

Euclidean distance-based methods for quantifying how distinct ensemble representations are for different stimuli are common and well documented (e.g., Chapin and Nicolelis, 1999; Stopfer et al., 2003; Daly et al., 2004b; Staudacher et al., 2009). Briefly, ED analysis was used to quantify the differences between odor-driven population responses across time (commonly referred to as population response trajectories). This allows us to determine how closely related ensemble response representations are across response time and as a function of odor and the context in which it was presented (Daly et al., 2004b). First, spike data were binned (5 ms) across a -1 to 5.5 s peri-stimulus time window. Spike counts were then z-score normalized based on a mean and standard deviation calculated from 3 s of spontaneous activity flanking each stimulus response (-1 to 0 s and 3.5 to 5.5 s). Each unit was treated as an independent coordinate vector and each bin represented the value of one coordinate in an N-dimensional space for its respective point in response time. N represents the number of units in the neural ensemble. We used entire population of 150 units from group one data. The Pythagorean Theorem was used to calculate the straight line distance between pairs of points, representing two different odors at the same point in response time (i.e., the corresponding time bins). As a point of reference we provide a threshold defined as ED values exceeding 2 SD from the mean ED for spontaneous activity. As ED values are based on normalized data, we parametrically compared distance values from all pair-wise comparisons between odorants as a function of the stimulation protocols to determine if between odor representations are statistically more distinct when odor was pulsed; this was performed using the GLM procedure in SAS.

## RESULTS

### A SUBPOPULATION OF NEURAL UNITS TRACK PULSED ODOR IN AN ODOR DEPENDENT MANNER

The primary goal of this study was to determine whether odor presented to the antennae in a manner simulating the periodic airflow that occurs during odor guided flight produces more distinct neural representations for odors from the AL; that is, are odors presented as pulse trains more easily discriminated as measured with population analytic techniques? To address this, we made extracellular recordings using tetrodes to measure the activity of AL neural units and LFPs *in vivo* while delivering temporally structured odorants directly to the animal’s antennae. The olfactometer was designed to rapidly and reliably time odor delivery with millisecond precision (**Figure 1**).

**FIGURE 2:**
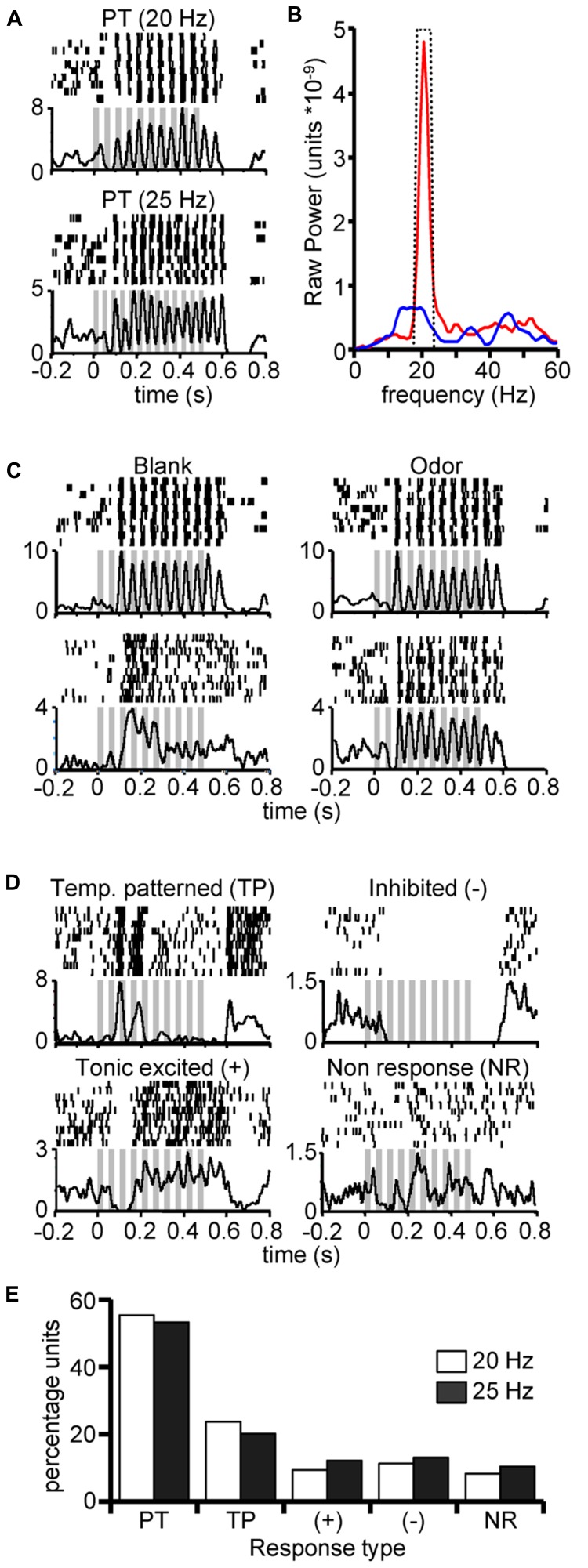
**Characterization of neural units exhibiting pulse tracking responses. (A)** Peri-stimulus rasters and corresponding histograms (below) from a single unit in response to 10 repeats of 500 ms 20 Hz (top) and 25 Hz (bottom) pulse trains of undiluted K8. **(B)** Typical results of power spectral density analysis of spiking in response to pulsed (red) and continuous (blue) stimulation for a same unit. Inset dashed line highlights the 4 Hz window centered on the pulsing frequency; integrated power measures were based on area within boundary and under curve. **(C)** Example peri-stimulus rasters from two units (rows) in responses to 20 Hz pulse trains elicited by blank (left) or odor (right) stimuli (empty odor cartridges) from units that were found to track pulsed stimuli. Odors were K10 and A9 for top and bottom panels, respectively. These highlight that unitary pulse tracking can occur in response to blanks but not all pulse tracking units track blank stimuli. **(D)** Examples of non-pulse tracking responses to 20 Hz pulse trains from units that were found to track pulsed stimuli (as shown in A and statistically confirmed in **(B)** to at least one odor. We defined four non-pulse tracking response classes: temporally patterned but not pulse tracking (TP), spike suppression (–), tonic excitatory response (+) and non-responsive (NR). Classification of response type was based on both a statistical change in firing rate and PSD analysis (see methods). Inset gray bars represent the odor presentation. Y-axis indicates trial-sum spike rates in a 1 ms binning window. **(E)** Percentage of unit responses (Y-axis) by response type (X-axis; defined above) and pulsing frequency. Results based only on units exhibiting a significant pulse tracking response to at least one odor (*N* = 36).

We first sought to determine whether molecular features, such as odor identity, impact the unitary spiking responses of AL neurons to undiluted pulsed odors. Across a group of 15 animals (*N* = 150 units), we observed a subpopulation of units that tracked pulsed stimuli, in many cases with discrete bursts of spikes. **Figure 2A** displays peri-stimulus rasters from one such unit as it responded to 20 Hz and a 25 Hz pulse trains and demonstrates that single units readily track each pulse with discrete bursts of spikes. The bursty nature of this unit during both spontaneous activity and in response to odor, as well as the brief spike suppression prior to the initial excitatory response are hallmark behaviors of PNs and was typical of the recorded units. It is possible that local interneurons were also recorded though there is no clear means to putatively identify them. Olfactory receptor neuron processes are too small to produce a detectable extracellular signal with our methods and thus are not represented in this study.

Next, to classify recorded units as pulse tracking, we individually calculated the PSD for each response to pulsed and continuous stimuli. Pulse tracking units typically produced a narrow-band peak at the 20 Hz pulsed frequency (**Figure 2B**) that was absent in the response to continuous odor (blue line). We then calculated the integrated power across a 4 Hz window centered at the pulsing frequency (**Figure 2B**, inset dashed line) and compared integrated power from pulsed and continuous stimuli using a Wilcoxon rank sum test (*p* < 0.05). This test was performed at two levels: one as an omnibus test across all odors to identify the number of pulse tracking units and the other a test within individual odors to determine the number of odors that each individual unit tracked. Consistent with our previous findings (Tripathy et al., 2010), we observed that 24 and 23% of all recorded units tracked pulsed odor at 20 and 25 Hz, respectively. Furthermore, 60% of pulse tracking units tracked pulsed blanks (cartridges with no odor) in addition to pulsed odor, whereas 40% of pulse tracking units tracked one or more odors but not blanks (**Figure 2C**). The observation that units track blanks in some cases but not others as occurs with odors, suggests that whether tracking occurs is dependent on the integration of both olfactory and non-olfactory cues.

Pulse tracking units did not universally pulse track to all odors; these non-tracking responses fell into four classes (**Figure 2D**). In many cases we observed excitatory responses to pulsed odor as indicated by a significant increase in peri-stimulus spike rate relative to pre stimulus spontaneous activity (*p* < 0.01) but did not produce significant power at the pulsing frequency (*p* > 0.05). For example, the upper left panel in **Figure 2D** displays the raster of a unit that exhibited significant pulse tracking to other stimuli but in this case produced a consistent burst pattern in response to the 20 Hz pulsed stimulus.

For all stimuli, we classified non-tracking responses such as these as temporally patterned (TP). We also observed significant tonic activation (+; **Figure 1C**, bottom left), inhibited (-; top right) and non-responsive (NR; bottom right). Overall, among the ~24% of units in these recordings which were identified as pulse tracking, they displayed significant power in the PSD only ~54% of the time (response distribution summary in **Figure 2E**). Thus, while pulse tracking is the most prevalent response type among this sub population of units, the fact that units do not always track implies that pulse tracking responses are odor dependent.

#### Unitary pulse tracking power increases with chain length

**FIGURE 3:**
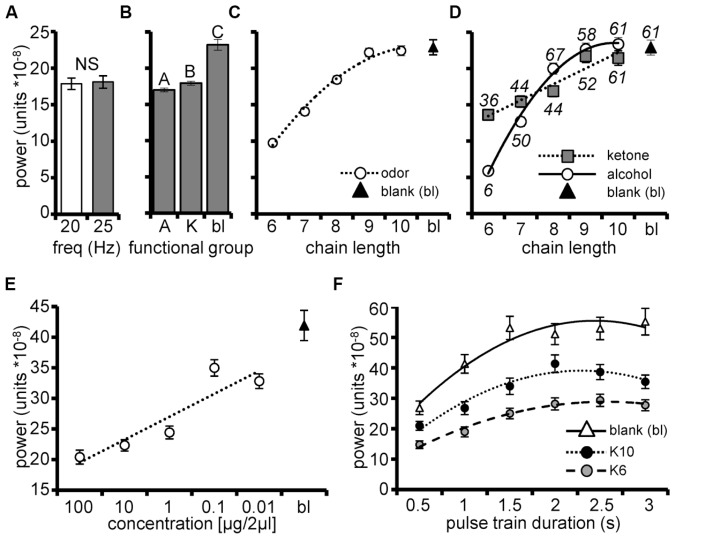
**Unitary pulse tracking is dependent on stimulus parameters**. Mean integrated pulse tacking power (Y-axis) among units as a function of frequency **(A)**, functional group (A = alcohol, K = ketone; bl = blank; **(B)**, and carbon chain length **(C)** for pulsed stimuli. Blank responses are included as a point of reference. Inset regression line is a second order polynomial highlighting the significant trend of increasing power as a function of chain length. **(D)** Mean power by the significant interaction of chain length and functional group from the subpopulation of units identified as pulse tracking (*N* = 36 units; 15 moths). Inset (above/below respective means) is the percentage of the pulse tracking cells that tracked a given stimulus. Inset are regression lines for ketones is a linear function and for alcohols is a second order polynomial function and highlight that the systematic trend for increasing pulse tracking power is a significantly steeper function for alcohols. **(E)** Mean integrated power as a function of odor concentration (*N* = 5 moths, 16 pulse tracking units). Inset linear regression indicates increasing power as a function of decreasing concentration. **(F)** Mean integrated pulse tracking power as a function of pulse train duration (*N* = 8 moths, 23 pulse tracking units). Results broken down by stimulus (blank, K10 and K6) to highlight that overall pulse tracking followed the same pattern as shown in **D** (i.e., the blank produced the greatest power followed by K10 then K6). Inset second order polynomial regressions indicate the same trend of increasing concentration as a function of increasing pulse train duration that asymptotes by 2 s.

In order to determine the nature of odor dependent responses, we statistically characterized integrated pulse tracking power as a function of the molecular features of the odors presented using GLM. Results of this model were significant (*F*_266,7433_ = 26.4; *p* < 0.0001; and explained 48% of the variance in integrated power. The main effect of pulse frequency was not significant (*p* = 0.9043) indicating that the AL was tolerant of variation in pulse frequency within the natural wing beat frequency range (**Figure 3A**). The main effects of functional group and carbon chain length were significant (*p* = 0.0012 and *p* < 0.0001, respectively). Blank odor cartridges (treated as separate category) elicited significantly greater power than odor belonging to either functional group (ketone or alcohol; **Figure 3B**). However, alcohols produced slightly but significantly lower power than the ketones. **Figure 3C** displays the significant effect of chain length on integrated power; the blank is inset as a comparison. The inset polynomial regression highlights this increase in power as a continuous function of increasing chain length. Note that responses to the blank stimuli produced the greatest raw power suggesting a non-olfactory component to the pulse trains.

In addition, the 2-way interaction between chain length and functional group was also significant (*p* < 0.001). **Figure 3D** displays mean integrated power among pulse tracking units (for 20 Hz) as a function of chain length now broken down by functional group. The inset second order polynomial function best described power as a function of increasing chain length for alcohols, while a linear function best explained the chain length relationship for ketones. This interaction simply suggests that the increase in power as a function of increasing chain length, progresses at different rates for the two moieties. Also inset in **Figure 3D** are the percentages of units which tracked each odor and indicate a 55% increase in the percentage of pulse tracking units as a function of increasing chain length; this too highlights the odor dependency of unitary pulse tracking. Finally, **Figure 3D** indicates that increasing power as a function of chain length is attributable to increased numbers of pulse tracking units.

As odor carbon chain length increases, odor volatility decreases, suggesting that the chain length effect may be attributable to concentration of odor that reaches the antennae. Therefore, in a separate set of recordings we tested how changes in stimulus concentration affected integrated power. In this case, odors were presented across a 4-log step dilution series ranging from 0.01 to 100 μg/2 μl odor in mineral oil. A blank stimulus and three ketones (K6, K7, and K8) were presented as 2 s, 20 Hz pulse trains; this longer duration optimized power measurements (see below). Based on psychophysical assays of odor detection (Daly et al., 2007) these odors are detected at 0.01 μg/2 μl and can be discriminated at 0.1 μg/2 μl. We again quantified the integrated pulse tracking power and modeled it as a function of stimulus concentration using GLM. In this case we hierarchically accounted for variance of all possible main effects and interactions prior to modeling concentration.

The overall statistical model explaining variation in the mean integrated power from the subpopulation of 16 pulse tracking units (from 5 moths) was significant (*F*_255,1024_ = 15.4; *p* < 0.0001; *R*^2^ = 0.79). After accounting for all other effects, the main effect of odor concentration was significant (*p* < 0.0001) with mean integrated power increasing as a linear function of decreasing concentration (**Figure 3E**). These results suggest that as odor concentration increases above detection and discrimination thresholds, AL units become less effective at tracking these odors.

Prolonged exposure to continuous stimuli results in rapid sensory adaptation and loss of odor guided behavior (Kennedy et al., 1981; Willis and Baker, 1984; Baker et al., 1985). Thus, we next determined the persistence of tracking when the AL was challenged with more prolonged pulse trains. In a final group of 8 moths (23 identified pulse tracking units), K6 and K10 and blanks were presented as 20 Hz pulse trains of durations ranging from 0.5 to 3.0 s; responses to continuous stimuli of equal durations were collected and used as statistical comparisons of significant pulse tracking (as described above).

The overall statistical model explaining variation in the mean integrated power generated by the subpopulation of 23 pulse tracking units was significant (*F*_413,1656_ = 23.1; *p* < 0.0001 and *R*^2^ = 0.85). After accounting for all significant factors previously described, we found a significant effect of pulse train duration (*p* < 0.0001) with pulse tracking improving as pulse train duration increases up to 2 s, regardless of odor tested (**Figure 3F**). Consistent with previous findings, blanks produced the greatest pulse tracking followed by K10 then K6 (**Figure 3C**).

### UNIT-LFP PHASE LOCKING IS STRONGLY DRIVEN BY TEMPORALLY STRUCTURED STIMULI

**FIGURE 4:**
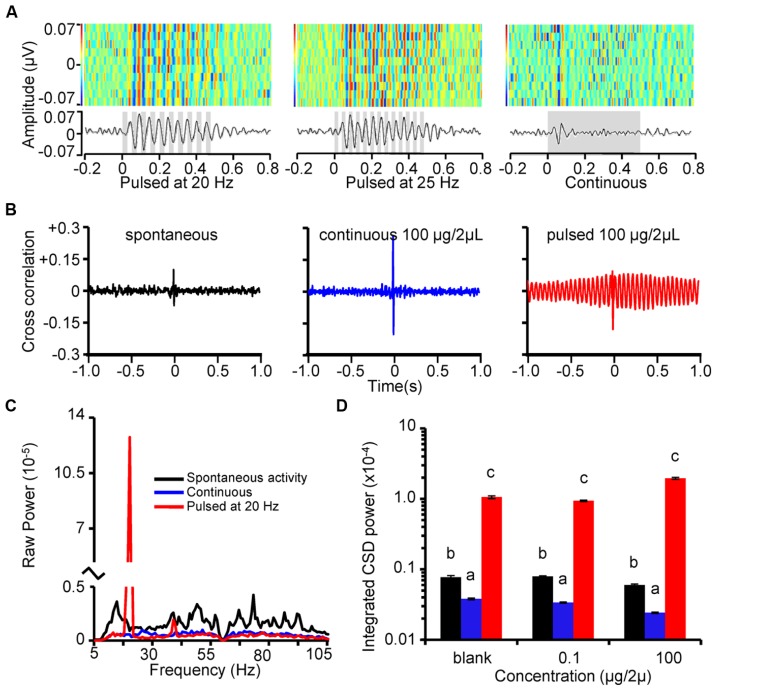
**Stimulus-driven LFP oscillations are strongly phase locked with unitary spiking. (A)** Rasterized peri-stimulus LFPs (top) and corresponding histograms (below) for 10 repeats of 500 ms odor stimulation (1-decanol; inset gray bars represent stimulus). Each LFP trace was band pass filtered with a 5–80 Hz and a 60 Hz notch then converted to a one-dimensional row of the raster. Heat-map color-code indicate voltage peaks (reds) and valleys (blues). All 10 responses are aligned by stimulus onset and stacked to create a single composite panel. Note that the vertical striping of red and blue (voltage peaks and valleys, respectively) for the pulsed stimuli indicates a near perfect alignment of oscillations. Peri-stimulus histograms (below) are binned at 1 ms. Results are from the same animal and recording site and are in response to 20 Hz (left), 25 Hz (center) pulse trains and a continuous stimulation (right). **(B)** Example cross correlograms between LFPs and units during spontaneous activity (left), and in response to K6 (100 μg in 2 μL mineral oil) presented as a continuous stimulus (center), or a 20 Hz pulse train (right). **(C)** Mean cross spectral density across all possible analyzed cross correlations. Results based on 80 individually calculated cross correlations across 4 pulse tracking units, 4 LFP recording sites, and 5 stimulus repeats. **(D)** Mean integrated CSD power in the cross correlations around the pulsing frequency. Results are color coded as in **B** and broken down as a function of odor concentration. Inset letters indicate significant differences between spontaneous, continuous, and pulsed CSD power at 20 Hz. Analysis was performed independently for each concentration. Note that relative to spontaneous activity, continuous odor stimulation significantly decreased power whereas pulsed stimulation significantly increased power; this was true at each stimulus concentration (Tukey’s HSD; *p* < 0.05).

The temporal relationship between LFP oscillations and spike timing has long been implicated as an odor identity encoding mechanism (for review see Laurent, 2002). We therefore asked whether the extrinsic, stimulus-driven oscillatory responses of units and LFP resulted in greater overall measures of unit-LFP phase locking relative to continuous stimuli. First, **Figure 4A** displays three peri-stimulus LFP heatmaps from one recording site in response to odor pulsed at 20 Hz (left), 25 Hz (center) and continuous stimuli (right), and demonstrates near perfect trial to trial coherence of the stimulus-driven oscillations. In response to the continuous stimulation on the other hand, LFP oscillations displayed a lack of regularity in oscillatory activity beyond an initial oscillation, which likely reflects the initial massed input to the AL.

To quantify the relationship between LFP oscillations and unit spiking activity we performed cross correlation analyses on these measures during spontaneous and activity evoked by continuous and pulsed stimuli. **Figure 4B** displays the cross correlation between an LFP (recording site 1; see **Figure 1**) with a single pulse tracking unit. Results are shown for spontaneous activity (left), and in response to K6 (100 μg/2 μL) presented continuously (center) or as a 20 Hz pulse train (right). In most cross correlations, a prominent spike in the correlation was present centered near *t* = 0 s, suggesting a high degree of coincident, possibly synchronous, activity between unitary spiking and LFP activity. In addition, we observed a strong oscillatory component in the unit-LFP cross correlation only when odor was pulsed; this is because pulsed stimuli entrains both unitary and LFP activity to a common response frequency.

Next we calculated the CSD of each response to pulsed and continuous stimuli as well as spontaneous activity sampled immediately preceding each stimulus. **Figure 4C** displays the averaged results of this analysis from a single moth and the four pulse tracking neurons identified in that recording. Results are based on all possible pair-wise cross correlations between each unit and all four LFP recordings. **Figure 4C** displays a single spike in power exclusively for the pulse-driven responses; this spike in power is precisely at the pulsing frequency and was an order of magnitude greater than the next highest peak. Consistent with our previous analysis of unit-LFP phase locking, continuous odor stimulation appears to decrease these measures as indicated by a relative lack of CSD power across the frequency spectrum (Tripathy et al., 2010). Results of a GLM explaining CSD power as function of spontaneous activity and odor-driven responses was significant (*F*_1547,56244_ = 27.6; *p* < 0.0001 and *R*^2^ = 0.43). We found a significant main effect of odor concentration (*p* < 0.001) and a significant difference in CSD power across the spontaneous, continuous and pulsed data (*p* < 0.0001). *Post hoc* analysis of integrated power in the CSD shown in **Figure 4D** highlights that pulsed stimuli on average produced an order of magnitude greater CSD power at 20 Hz than did spontaneous activity or continuous stimuli. This means that the degree to which unitary and LFP coordination is far more strongly controlled by the temporal features of the stimulus than the intrinsic oscillatory dynamics of the AL. Again, we observed a consistent and significant decrease in distributed oscillatory power for the continuous stimulation relative to spontaneous activity, indicating that prolonged continuous odor presentation in fact decreases unit-LFP phase locking (Daly et al., 2011).

### PULSED ODOR INCREASES SEPARATION OF ODOR REPRESENTATIONS IN THE AL

While pulse tracking responses are odor-identity dependent, this finding alone does not establish whether representations for odors across the population are statistically more distinct as compared to representations produced by continuous stimuli. To address this question, population-based ED analyses were used (e.g., Chapin and Nicolelis, 1999; Stopfer et al., 2003; Daly et al., 2004b; Staudacher et al., 2009) to measure the separation or discriminability between population spiking responses to different stimuli. For this analysis, we pooled units across animals where *N* = 150 represents the number of total units in the pooled population. Here, we calculated the ED between population spiking responses to different odorants and stimulation protocols by (1) counting each unit’s number of spikes in a given time bin (bin size = 5 ms), z-score normalizing these counts, and (2) concatenating each unit’s normalized spike count in a time bin into a 150-dimensional vector, which represents the population’s spiking activity in a given time bin. Thus EDs were calculated by applying the Pythagorean Theorem to compute the straight line distance between pairs of points in the 150-dimensional space, each point representing the same peri-stimulus bin in response time for two comparison stimuli. EDs were calculated for each bin in response time for each comparable pair of stimuli.

Using this ED analysis, we first sought to determine whether odors have different neural representations when pulsed versus delivered continuously (i.e., a *within-odor* comparison of different stimulation protocols). For this analysis, all possible pair-wise comparisons of distance between pulsed and continuous stimuli for the same odorant were calculated (for example, comparing all responses to K6 when delivered pulsed versus continuous). We next sought to determine whether the separation between odor representations was increased by pulsing odor stimuli versus presenting continuous stimuli (i.e., a *between-odor* comparison within a stimulation protocol; for example, whether responses to K6 versus A6 are more discriminable when pulsed versus continuous delivered). Here all possible pair-wise between odor comparisons of pulsed or continuous stimuli were compared.

**FIGURE 5:**
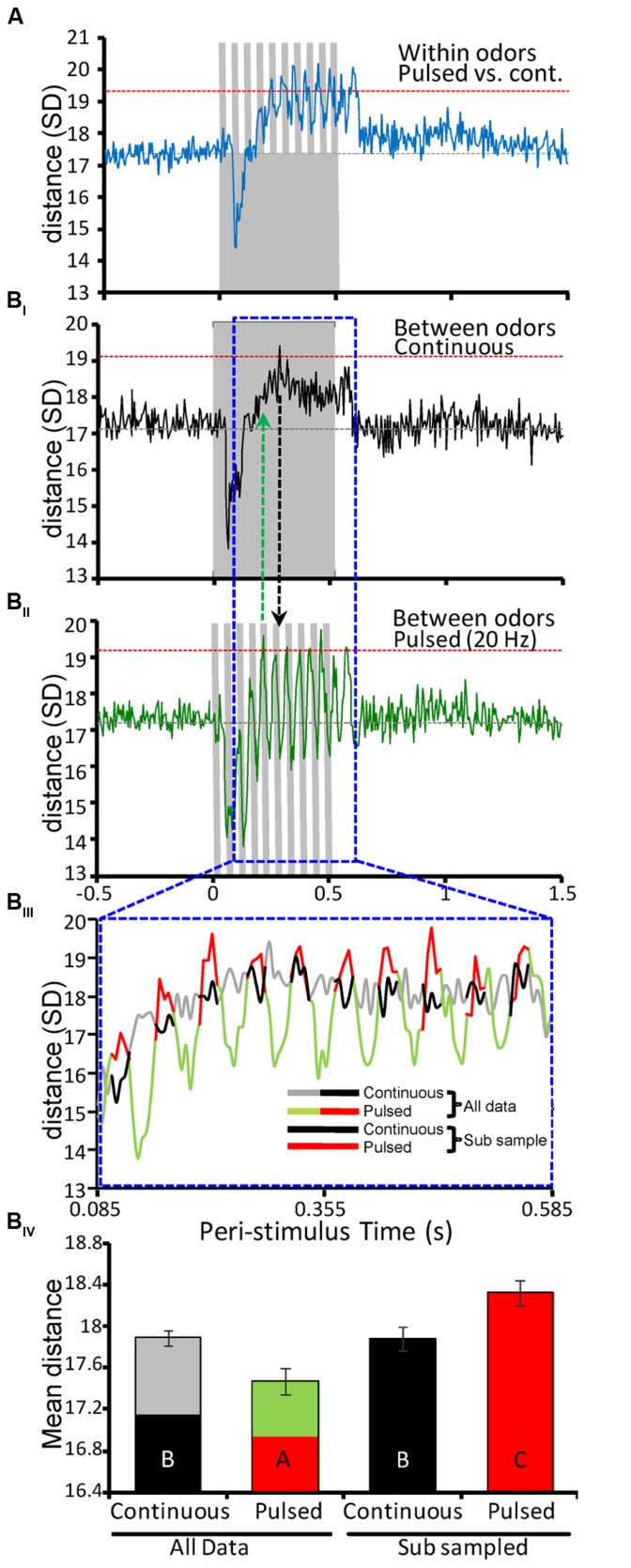
**Antennal lobe odor representations are more distinct when odor is pulsed. (A)** Mean within-odor Euclidean distance (ED) between comparisons of population responses elicited by the same odor presented as either 20 Hz pulse trains or as continuous stimuli. All stimuli were 500 ms in duration (inset gray bars). Analysis was performed on individual stimulus repeats using a 5 ms binning window and results were then averaged across repeats for all odors (*N* = 10 per odor/stimulation protocol). Results are based on the entire population of recorded units (*N* = 150). Inset gray horizontal line represents the mean spontaneous ED value. Inset horizontal red lines represent ± 2 standard deviations (SDs) from that mean distance value. **(B)** Results of between-odor ED analysis for only the continuous **(B**_I_) or 20 Hz pulsed **(B**_II_) stimuli. Analysis based on all possible pair-wise comparisons between the different odors across the recorded population (*N* = 4500; 10 odors, 10 repeats, 150 units). **(B**_III_) Expanded and superimposed comparison of ED measures for an odor-driven response window (0.085–0.595 ms post-stimulus onset; highlighted above with inset dashed blue frame). The ED measures for the pulsed (green/red) and continuous (gray/black) are color contrasted to highlight specific statistical comparisons of time-averaged distance in **(B**_IV_). Here the excitatory phase of each pulse-driven response is highlighted in red. Every other 25 ms in response time was sub-sampled, which reflects the 25:25 ms on:off duty cycle. For comparison the corresponding time periods from the continuous stimuli was also sub-sampled (in solid black). **(B**_IV_) Mean ED for pulsed and continuous stimuli. Here the four means represent the time-averaged ED for either the entire response window (All data) or the sub-sampled data points highlighted in **B**_III_. Inset letters indicate significant differences between means (Student’s *t*-test; *p* < 0.05). Comparison of integrated ED for the entire response window indicates that continuous stimuli produce significantly more distinct odor representations. By comparison, when only the excitatory component of the pulse responses is considered (i.e., the sub-sampled data) pulsed stimuli lead to significantly better separation of odor representations than continuous stimuli. All error bars indicate standard error of the mean (SEM).

**Figure 5A** displays the mean ED by time for all pair-wise within-odor comparisons of pulsed versus continuous stimuli. The initial phase of the response is demarked by a sharp decrease in distance between responses, which initiated within ~50 ms and reached their lowest values by ~75 ms. Thus, for this initial period the responses to the same odor presented different ways was highly similar. This dip in distance is consistent across prior multiunit studies (Daly et al., 2004b; Staudacher et al., 2009) and is attributable to early, I_1_ inhibition, which is exclusively expressed in PNs (Christensen et al., 1998). Thus, because the initial response (spike suppression) is common to all odor-driven responses (i.e., both pulsed and continuous), distance is minimized. After this initial inhibitory phase, EDs increased and maximized by ~150 ms and then oscillated at the pulse train frequency. Importantly, the responses to continuous and pulsed stimuli during this excitatory phase can be characterized as dissimilar based on the observation that on a periodic timescale, ED became significantly different as indicated by a crossing of the threshold of 2 standard deviations (**Figure 5A** inset red line). At ~600 ms after stimulus onset EDs dropped sharply, returning to baseline. These results establish that odor representations are significantly different as a function of the manner in which they are experienced.

The final and most important aspect of this study is the impact of temporal stimulus structure on representation separation for different odorants. In other words, does the temporal structure produced by the pulsed versus continuous stimulation protocols result in different odorants having more separable neural population responses? Relative to continuous stimuli (**Figure 5B**_I_), different odors are more quickly and effectively separated when odor is pulsed (**Figure 5B**_II_). This analysis was based on all possible pair-wise between-odor comparisons. As in **Figure 5A**, both stimulation protocols show the hallmark effect of I_1_ inhibition at precisely the same time. However, whereas between-odor EDs for continuous stimuli reach an optimum by 280 ms of stimulus onset, the same population achieves peak distance within only 210 ms when odor is pulsed (**Figure 5B**_II_); inset vertical lines highlight this difference in latency to ED peak. Furthermore, ED measures for pulsed stimuli produced oscillating peaks, six of which crossed the threshold of 2 standard deviations. These results suggest that pulsed odors provide more persistently separated representations on a periodic timescale, whereas ED measures for prolonged continuous stimuli appear to attenuate after reaching an initial peak, possibly as a result of adaptation.

Lastly, we sought to calculate a single measure defining the representation separation for between-odor comparisons as a function of stimulation protocol. Thus, we averaged ED values across a temporal window encompassing the excitatory component of all responses, ranging from 0.085 to 0.585 ms (i.e., 100 total 5 ms time bins; **Figure 5B**_III_). Keeping in mind that pulsed stimuli only produce periodic excitatory responses within this window, we analyzed time-averaged ED distances in two ways. First, we compared mean distances overall (i.e., utilizing all ED values from all 100 time bins for each stimulation protocol). Next, we compared mean distance as a function of only the excitatory epochs in the pulsed ED. As highlighted in **Figure 5B**_III_, we sub-sampled the excitatory responses of the pulsed ED measures (red traces), and corresponding data from the continuous ED measures (black traces) for statistical analysis. This analysis was based on variation in mean ED. The sub-sampling interval was defined as the first 5 consecutive values (25 ms, given 5 ms bins) on the rising phase of the first pulse response (i.e., **Figure 5B**_III_ red traces) then dropping the following 5 values (**Figure 5B**_III_ green traces), and so on; the pattern of every other 5 values reflects the 25:25 ms on:off duty cycle (pulsed at 20 Hz) and yields 50 mean distance values for analysis. The same sub sampling method was used on the continuous data as well for comparative purposes. **Figure 5B**_IV_ represents the time-averaged EDs for continuous and 20 Hz stimuli computed using either the entire temporal window (all data) or the sub-sampled data considering only excitatory epochs as described above. Statistical comparison of these mean values indicates that the excitatory component of the pulsed response lead to significantly greater distance between odors than continuous stimuli (inset letters, Student’s *t*-test; *p* < 0.05). That is, simulating the natural flow dynamics generated during odor guided flight produces more distinctive and discriminable AL representations. As observed in other sensory systems, periodic input thus results in statistically more distinct neural representations of odors relative to prolonged continuous stimuli.

## DISCUSSION

It has long been known that odor plumes are complex, containing a “wispy” filamentous spatiotemporal structure (Murlis and Jones, 1981; Mafraneto and Carde, 1994; Carde, 1996; Murlis et al., 2000). Studies of the time varying concentration of odor plumes in wind tunnel and field indicate that the distribution of stimulus durations a moth might encounter is on the order of 100 to about 500 ms, though this clearly is dependent on several environmental factors (Murlis et al., 2000). It is within the context of this chemical ecology that moths actively and periodically sample their olfactory environment, discretizing this chemical ecology into spatiotemporal “moments.” Studies of wing kinematics demonstrate that as a moth transitions from high speed flight to odor guided and hovering flight, the orientation of the body, wing stroke path, and orientation change, bringing the wings closer to the antennae (Willmott and Ellington, 1997a, b). Given that it is necessary for the flightless moth *B. mori* to have and beat its wings during plume tracking behavior in order to find an odor source (Obara, 1979), it stands to reason that flying insects might also exploit the physical forces induced by the beating wing to actively sample odors as well (Schneider, 1964). Recent biomechanical, modeling, behavioral and physiological studies suggest that the act of wing beating is exploited as an active olfactory sampling strategy (Loudon and Koehl, 2000; Bau et al., 2005; Sane, 2006; Sane and Jacobson, 2006; Tripathy et al., 2010). Thus we sought to characterize and compare physiological responses to fast periodic stimuli, typical of wing beating during natural odor guided flight behavior, relative to prolonged and continuous stimuli, more typically used in laboratory studies of insect olfaction. Similar approaches have been taken previously but used lower pulse frequencies and longer pulse durations (Brown et al., 2005; Geffen et al., 2009). Our results indicate that pulse train stimuli drive the phase locked entrainment of LFPs as well as a population of ~25% of AL units at higher frequencies than previously described. Importantly, analyses of population responses indicated that the spatiotemporal details of brief and periodic responses were significantly different from responses to the same odors presented as continuous stimuli.

Intermittent stimuli improve representations in sensory systems. For example, microsaccades are a constant rhythmic movement of the eye observed during visual target fixation. It is thought that these rhythmic movements are a mechanism for counteracting sensory adaptation (Martinez-Conde et al., 2006). Consistent with visual processing, brief pulse responses resulted in significantly greater separation between odor representations than continuous stimuli. Furthermore, pulsed odor representations were more quickly and easily discriminated by the ED analysis. These results likely underlie psychophysical results indicating lowered detection thresholds when odor is pulsed (Daly et al., 2013). The fact that continuous odors produced an attenuated ED measure over time while pulsed odor did not seems to support the conclusion that periodic olfactory input may serve to counteract adaptation.

“Olfactory” responses in the absence of an apparent odor are commonly observed in mammals (Walsh, 1956; Macrides and Chorover, 1972; Ravel et al., 1987) and in several moth species including *Spodoptera littoralis* (Anton and Hansson, 1995), *Heliothis virescens* (Galizia et al., 2000), *B. mori* (Kanzaki and Shibuya, 1986), and *M. sexta* (Kanzaki et al., 1989; Staudacher et al., 2009). Similar blank responses are observed in other insect species as well (Zeiner and Tichy, 1998). Consistent with these observations, we observed that blank cartridges, in addition to less volatile odors and odors presented at lower concentrations all entrained unitary responses best. The highly simplified olfactometer we used makes odor contamination an unlikely explanation. Furthermore, it is unlikely that the blank pulse response is related to oscillations in humidity or temperature as all air passing through the output nozzle is supplied from a single source that is only split upon entering the 3-way valve (see **Figure 1A**). Hotwire anemometry on the other hand has established that the 3-way valve used in our olfactometer produces a highly stereotypic flow artifact that could provide a mechanosensory cue (Daly et al., 2013). Thus the parsimonious explanation is that responses to empty cartridges are due to mechanical “wind” effects that have been observed in many systems. Placed within the context of pulsed odor, responses to pulsed blanks may therefore reflect an underlying mechanism that integrates olfactory and mechanosensory information to enhance the temporal resolution of olfactory processing. This suggests that wing beating may superimpose a rhythm that facilitates the temporal resolution at which odor stimuli can be sampled; our demonstration of increased separation of odor representations when pulsed supports this proposition. Thus, we concur with others that airflow responses are the most likely explanation, and should be considered an integral and necessary part of the olfactory experience (Mainland and Sobel, 2006; Wachowiak, 2011). However, while the most likely explanation appears to be mechanosensory, the source of the response to this within the AL remains unknown. Our current understanding is that mechanosensory input from the antenna bypasses the AL and ramifies the antennal mechanosensory and motor center (Homberg et al., 1989). There is no anatomical data that we are aware of showing projections from the antennal mechanosensory and motor center back to the AL. This suggests that the source of the blank response is from the olfactory receptors themselves. Future studies should focus on attempting to determine the source of non-olfactory responses in the AL, using methods that uncouple putative mechanosensory and odor cues while maintaining the high temporal frequencies used herein.

Finally, it is interesting that although pulsed blanks caused the greatest amount of AL unitary entrainment, pulsed blanks are least likely to cause a false positive conditioned response in behavioral assays of odor detection (Daly et al., 2013). We speculate that perhaps the periodic blank response is an expected component of the primary olfactory function, which may be filtered in downstream processing centers.

Different odor encoding models have been proposed to explain how neural circuits in primary olfactory centers establish identity codes for odors for subsequent readout in downstream centers. Some have argued that odor identity is encoded by transient synchronization of spikes across a dynamic assembly of neurons on a periodic timescale spanning several hundreds of milliseconds and established by intrinsically generated subthreshold LFP oscillations (Laurent, 2002). This oscillatory mechanism results in a temporally structured output code from primary olfactory networks that are read out downstream by the mushroom bodies. They argue that a temporal coding scheme provides better discrimination at the cost of requiring more time to encode. However, the transient oscillatory synchronization does not appear to play a role in odor discrimination in *M. sexta* (Mwilaria et al., 2008; Daly et al., 2011). Alternatively, it has been proposed that odor drives “onset” synchronization of the output response from primary olfactory centers allowing the AL to track the time varying concentration of the plume structure (Christensen et al., 2003; Lei et al., 2009). We have argued that odor dependent representations are correlated to a sequence of onset latencies from different subsets of synchronously active AL outputs that evolve over and are optimized within <150 ms (Daly et al., 2004b; Staudacher et al., 2009). We highlight that although this temporal evolution is interrupted by temporal structuring imposed by simulated wing beating (relative to the 100 ms single odor pulses we used in prior work), the ED analysis presented herein also takes the same approximate time to optimize as we have previously described, ~150 ms from the start of the response. The longer continuous stimulations appear to lengthen the time to peak ED. Thus, our current data appears consistent with our previously published findings suggesting a limited role for time in the encoding of odor identity but a major role for the encoding the time varying structure of the stimulus.

## CONCLUSION

This study establishes that representations for different odors become more distinct when odors are presented as very brief and intermittent pulses as opposed to prolonged continuous odor stimulation; this may be facilitated by the non-olfactory component of the response. Brief and intermittent pulses simulate the natural periodicity of wing beating during odor guided flight and therefore represent a more “natural” and biologically relevant stimulus. When odor is presented in this manner, odor representations also optimize more quickly (within 3 pulses) and are more persistent, showing little if any adaptation. Our findings support the notion that the olfactory system of this moth is optimized to process odor signals within a timeframe defined at least in part by wing beating, a result that is not observable with relatively prolonged stimulations as is commonly used in the laboratory setting. We therefore emphasize the importance of using more “natural” brief stimuli that are appropriately shaped to match the species specific chemical ecology, as this might help reconcile discrepancies between different models of odor identity encoding.

## Conflict of Interest Statement

The authors declare that the research was conducted in the absence of any commercial or financial relationships that could be construed as a potential conflict of interest.
